# Match Analysis of Soccer Refereeing Using Spatiotemporal Data: A Case Study

**DOI:** 10.3390/s21072541

**Published:** 2021-04-05

**Authors:** Bruno Gonçalves, Diogo Coutinho, Bruno Travassos, João Brito, Pedro Figueiredo

**Affiliations:** 1 Departamento de Desporto e Saúde, Escola de Saúde e Desenvolvimento Humano, Universidade de Évora, 7000-812 Évora, Portugal; 2 Comprehensive Health Research Centre (CHRC), Universidade de Évora, 7000-812 Évora, Portugal; 3 Portugal Football School, Portuguese Football Federation, 1495-433 Oeiras, Portugal; brunotravassos@hotmail.com (B.T.); joao.brito@fpf.pt (J.B.); pedro.figueiredo@fpf.pt (P.F.); 4 Research Center in Sports Sciences, Health Sciences and Human Development (CIDESD), University of Trás-os-Montes and Alto Douro, 5001-801 Vila Real, Portugal; diogoamcoutinho@gmail.com; 5 Research Center in Sports Sciences, Health Sciences and Human Development (CIDESD), University of Beira Interior, 6201-001 Covilhã, Portugal; 6 Research Center in Sports Sciences, Health Sciences and Human Development (CIDESD), University Institute of Maia (ISMAI), 4475-690 Maia, Portugal

**Keywords:** performance analysis, refereeing, positional data, tracking systems, tactical positioning

## Abstract

This case study explored how spatiotemporal data can develop key metrics to evaluate and understand elite soccer referees’ performance during one elite soccer match. The dynamic position of players from both teams, the ball and three elite referees allowed to capture the following performance metrics: (i) assistant referees: alignment with the second last defender; (ii) referee: referee diagonal movement—a position density was computed and a principal component analysis was carried to identify the directions of greatest variability; and (iii) referee: assessing the distance from the referee to the ball. All computations were processed when the ball was in-play and separated by 1st and 2nd halves. The first metric showed an alignment lower than 1 m between the assistant referee and the second last defender. The second metric showed that in the 1st half, the referee position ellipsis area was 548 m^2^, which increased during the 2nd half (671 m^2^). The third metric showed an increase in the distance from the referee to the ball and >80% of the distance between 5–30 m during the 2nd half. The findings may be used as a starting point to elaborate normative behavior models from the referee’s movement performance in soccer.

## 1. Introduction

Soccer is a team sport where two teams dynamically compete in space and time to gain an advantage over the opposing team; the game is mediated by the referee and the assistant referees that ensure players perform under the rules [1]. Though, the referees need adequate physical performance, knowledge, and decision-making regarding the laws of the game. Actually, referees must ensure the application of the laws of the game coherently to the game dynamics and in a uniform way to both teams [2].

The first body of research exploring soccer referees’ performance was focused on physical performance. In general, soccer referees cover between 9 to 13 km during a match, from which ~17% consists in high-intensity running (i.e., from 15 to 18 km/h) [3]. Assistant referees cover ~6760 m, from which ~1540 m result from sidewards movements while attempting to follow the ball and defensive players positioning [4,5]. However, both for referees and assistant referees, the amount of distance covered is affected by contextual factors, such as competitive standard [3,5]. Overall, these findings provide vital information to assist in designing and planning training interventions that allow soccer referees and assistant referees to cope with the match physical demands.

Less is known regarding the contextualization of referees’ displacements to accomplish technical and tactical match requirements. The research scope has recently shifted from the physical perspective towards understanding which factors mediate game management and referees’ decision-making. Notably, expert referees show better anticipatory strategies and higher intervention speed, supported by better positioning on the field according to the game’s flow [6,7]. In fact, to apply the laws of the game, the referee must permanently move in the pitch to ensure the best view to perceive the match incidents and identify the relevant cues for proper decision-making [3].

Additionally, the role of the two assistant referees should be acknowledged, as they attempt to help the referee judge playing actions and identify possible offside positions from the offensive team [8]. Although assistant referees have a key role during the game, studies that focus on analyzing their performance and the teamwork developed with the referee are still scarce [3].

Previous research related to the soccer referees’ teamwork revealed that high-level referees cooperate more effectively with the assistant referees than less experienced referees, reducing the distance and grey zones between them and involving them more in the decisions [9]. Accordingly, Helsen and Bultynck [7] reported that 64% of all refereeing decisions are based on teamwork. Thus, proper positioning on the pitch seems essential for making the right decisions collectively and decreasing the error rate. For instance, previous research has reported that the distance to the ball, the angle of viewing, and the referee’s velocity at the moment of decision are crucial to decrease the error in the process of judgment and decision-making between referees and assistant referees [10]. Accordingly, referees’ appropriate decisions seem to emerge from distances between 11 and 25 m [1,8]. However, the findings from these studies did only consider foul play incidents. More recently, another study explored not only the possible whistled situations but also incorrectly non-whistled situations. The referees were more likely to whistle at medium distances (i.e., 10 to 15 m) while refrained from whistling at lower distances (i.e., 0–5 m) [11]. However, when considering potential penalty situations, referees seemed to make more proper decisions in distances below 10 m than longer distances [12].

Altogether, there is still some inconstancy regarding appropriate referee positioning, and further research is required to attenuate such discrepancies. More than understanding central distances, it might be essential to understand the referee’s spatial area and the spatial relationship between assistant referees and teams’ defensive lines, providing additional insights regarding refereeing positioning. However, to the best of our knowledge, no study so far has addressed this issue.

Despite the supportive role of assistant referees over the match, offside call decisions may also impact the match outcome. An error rate of 20% to 26% during offside decisions has been reported [13]. This percentage of errors seemed to be dependent on the assistant referees’ angles of view and positioning. In fact, the assistant referee’s position concerning the attacker and the second last defender has a crucial effect on their decision [14]. In accordance, the two main hypotheses explaining assistant referees’ wrong decisions related to their incorrect positioning were: (i) optical-error hypothesis, which refers that the referee may raise the flag while the attacker is not offside (flag error) or not raising it while they are [15]; and (ii) flash-lag effect that consists of a visual illusion in which an object is perceived to be in a more advanced position compared with its real position [16]. To decrease such errors, the visual angle has been suggested as crucial information to make the situations easier to judge [17]. A previous report explored the visual angle from the assistant referee to the set play, measured through the position of the attacker (i.e., the player in possession), the assistant referee, and the offside (i.e., defined by the second last defender or the potential receiver if being offside). For that purpose, the following visual angles ranges were considered: 0–15°; 16–30°; 31–45°; 46–60°; 61–75°; and >75°; the authors found a lower percentage of errors when the visual angle ranged between 46–60° [8]. Regarding the distance to the offside line, it has been considered that the assistant referee was in line with the offside line when presenting a positioning lower than 0.20 m from this line [13]. Though, it should be noted that assistant referees can maintain such alignment only by 14% of the time [8], and most of the studies were only focused on offside calls.

Overall, the referees’ decisions seem to depend upon their positioning on the pitch and the ball location [2,8,11,14]. Therefore, it is essential to understand how referees move depending on their positioning on the pitch, the ball and the assistant referees, aiming to cover the best possible match incidents and make correct decisions. Accordingly, recent literature has been using the information derived from tracking systems to capture the players’ performance across the match based on spatiotemporal metrics [18], such as heatmaps [19], ellipsis areas [20], and distances between players [21]. Therefore, a more comprehensive understanding of referees and assistant referees positioning concerning the ball and players’ movements can be achieved using similar methods.

The present case study is a first attempt to explore new metrics to understand soccer referees’ positioning during match-play, the relation with the ball displacement, and the assistant referee’s relation regardless of the teams’ defensive line.

## 2. Materials and Methods

One international soccer match from elite adult male players was used for analysis. Data relate to one elite referee’s position, two assistant elite referees (AREF), all players and the ball. For that purpose, the TRACAB Optical Image Tracking System (Chyronhego, New York, NY, USA; https://chyronhego.com) captures and transforms the coordinates into a two-dimensional plane, with a frequency of 25 Hz. Supported by eight super-HD cameras, the players are identified based on their movements, shape, and color information [22]. Each camera creates a stitched panoramic view, allowing triangulation of the players and the ball due to a stereoscopic view [23]. Based on its accuracy (i.e., it has been showing a maximum delay of just three frames for all moving objects), several professional soccer leagues have been using it (e.g., English Premier League, German Bundesliga, or the Spanish La Liga) as well as UEFA Champions League and FIFA international tournaments.

Similarly, recent research has used the TRACAB system to explore physical performance according to age groups [22] and half variations [24], offensive performance in football [25], how the quality of opposition impact the spatial-temporal features of individual ball possessions [26], or even to understand the risk of contact exposure during football matches in the context of the COVID-19 pandemic [27]. This system has also provided reliable results compared with other reference systems (VICON motion capture system) [23]. When considering the total distance travelled and peak speed, only trivial deviations were found according to this reference system (0.42 ± 0.60% and <0.5%, respectively). Moreover, the root means square error was 0.09 m for position measurement, 0.09 m∙s^−1^ concerning the instantaneous speed, and 0.026 m∙s^−2^ in accelerations.

### 2.1. Metrics, Processing Steps, and Analysis

Three data-driven approaches were carried, and the following performance metrics were developed.

### 2.2. Referee Diagonal Displacement

Referee position density was computed, and a principal component analysis (PCA) was performed to identify the displacement variability, with two orthogonal segments centered on the referee’s mean position.

A position density represented by a heat map (based on the referee’s x and y position coordinates across the match) was computed for both halves. The colored scatterplot represents a continuous 2D distribution, in which the dot sizes correspond to the swarm points density. The algorithm uses the x and y coordinates from the referee as the same size vectors, the local radii parameter surrounding every data point, and the weighted dots for the corresponding area parameter (for algorithm computation, see Sundqvist [28]). These procedures allow specifying pitch zones where the referee spent the most time (i.e., positioning density) and provide a visual perspective of the referee displacement across the game.

A PCA was applied to identify the referee position variability during the match, using the referee’s mean position on the pitch. The dataset consisted of one matrix for the referee (x(i), y(i)), where x(i) and y(i) represent the coordinates on the pitch during the match, with i = 1,...,N, where N is the match time. Two orthogonal segments were centered on the referee’s mean position, and the segments’ directions were driven by the eigenvectors of the PCA [29]. The results of this processing step were plotted within the referee position density heat map.

### 2.3. Distance from the Referee to the Ball

The distance from the referee to the ball was computed by the norm between the vectors using the following equation:

$$Distance\left(a_{x_{\left(t\right)},y_{\left(t\right)}},b_{x_{\left(t\right)},y_{\left(t\right)}}\right)\;=\;\sqrt{{\left(a_{x_{\left(t\right)}}\;-\;b_{x_{\left(t\right)}}\right)}^{2}\;+\;{\left(a_{y_{\left(t\right)}}\;-\;b_{y_{\left(t\right)}}\right)}^{2}},$$

where *a* is the referee, *x* and *y* are the coordinates for both goal-to-goal and pitch width directions, respectively, *t* is the time, and *b* is the ball. Moreover, distances were also analyzed according to the amount of variability expressed by the coefficient of variation.

### 2.4. Alignment Difference of the Assistant Referee with the 2nd Last Defender (Offside Line)

**Figure 1:**
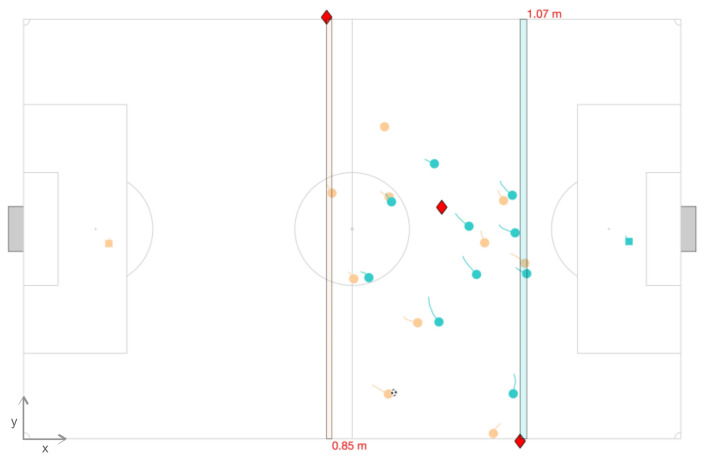
Match frame animation. The referee and the assistant referees are plotted as red diamonds. The distance depicts the alignment of the assistant referee with the 2nd last defender. For example, the assistant referee on the right is 1.07 m misaligned with the 2nd last defender (offside line).

This metric was calculated based on the difference between the x-coordinate position of the assistant referee (AREF) (goal-to-goal direction) and the x-coordinate position of the 2nd last defender (from the defending team of the AREF half-pitch) according to the formula:

$$Alignment\left(a_{x_{\left(t\right)},},b_{x_{\left(t\right)}}\right)\;=\;a_{x_{\left(t\right)}}\;-\;b_{x_{\left(t\right)}},\;$$

where *a* is the AREF, *x* is coordinate for goal-to-goal direction, *t* is the time, and *b* is the 2nd last defender (see Figure 1). The computation excluded when the 2nd last defender moved to the offensive pitch.

Data from the 1st and 2nd halves were independently treated and processed when the ball was in-play (i.e., all the stoppages were excluded from the analysis). The calculations and visualizations were performed in Matlab^®^ (The MathWorks Inc., Natick, MA, USA). The descriptive statistics and the PCA were computed with the SPSS software V24.0 (IBM SPSS Statistics for Windows, IBM Corp, Armonk, NY, USA).

## 3. Results

### 3.1. Referee Diagonal Displacement

**Figure 2:**
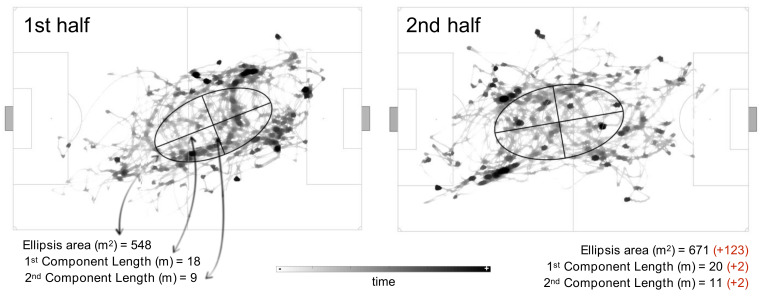
Referee diagonal movement. The grey heat map represents the referee position density (darker areas represent more time spent), and the ellipse shows the referee position variability during each half (computed by the principal component analysis).

Figure 2 shows the referee position density and the displacement variability (PCA outcomes). The referee displacement showed that, in the 1st half, the position ellipsis area was 548 m^2^, with the 1st principal component (length position) of 18 m, and with the 2nd principal component (width position) of 9 m. In the 2nd half, those values increased 123 m^2^, to 671 m^2^ for the ellipsis area and 2 m in both the 1st and 2nd component to 20 and 11 m, respectively, suggesting lower diagonal movements and shorter displacements around the center of the ellipsis.

### 3.2. Distance from the Referee to the Ball

**Figure 3:**
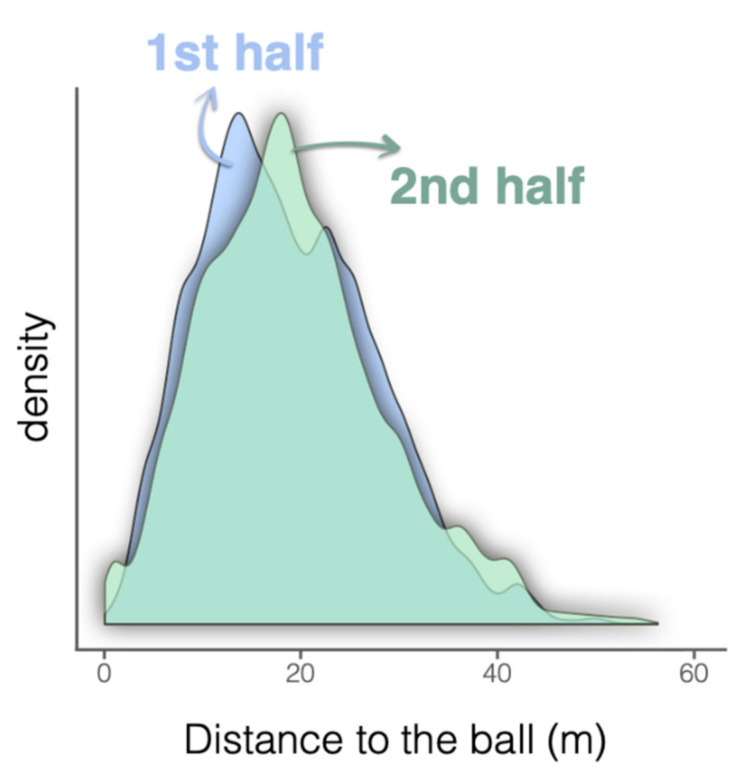
Density distribution of the distance from the referee to the ball for each half.

Figure 3 and Table 1 characterize the distance from the referee to the ball for each half. The referee spent >80% of the time within the distance standing between 5–30 m. The values slightly increased during the 2nd half (from 17.66 to 18.25 m, median) with approximately 50% of the coefficient of variation and ~0.5 of skewness. The overlap of two density histograms (i.e., both halves) was 89.1%.

### 3.3. Alignment Difference of the AREF with the 2nd Last Defender (Offside Line)

**Figure 4:**
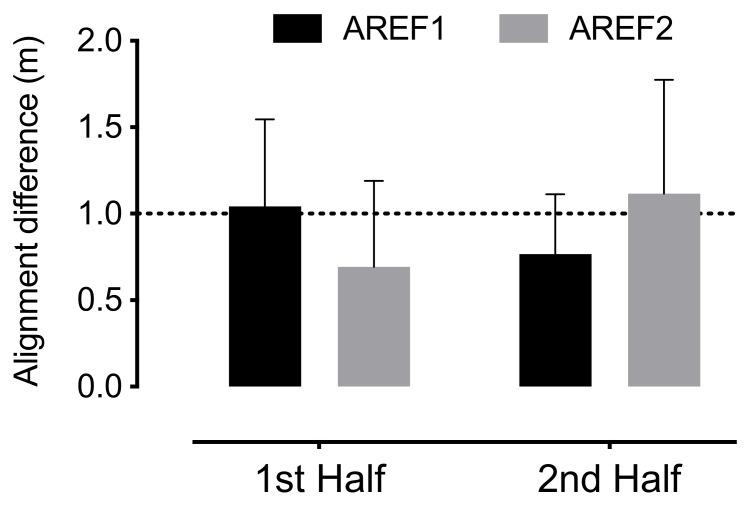
Alignment of the assistance referees (AREF) with the 2nd last defender.

Figure 4 depicts the alignment difference between the AREF and the 2nd last defender during the match. The average values were approximately lower than 1 m. The results changed from the 1st to the 2nd half: in the 1st half, the alignment difference for AREF1 was higher than for the AREF2 (AREF1 = 1.04 ± 0.51 m, AREF2 = 0.77 ± 0.35 m), while in the 2nd half, the alignment was lower for the AREF1 (0.70 ± 0.50 m).

## 4. Discussion

The present study aimed to explore new metrics to understand soccer referees’ in-match positioning, the relation with the ball displacement, and the assistant referees’ relation regardless of the teams’ last defensive line. It was also explored how the referees’ performances varied from the 1st to the 2nd half. In general, the results showed that the referee maintained a stable distance to the ball throughout the game. However, lower diagonal movements followed by a slight increase in the distance to the ball during the 2nd half were registered compared to the 1st half. Regarding the assistant referees, the results revealed alignments with the 2nd last defender of ~1 m, suggesting a high level of movement coordination of assistant referees with the last player from the defensive line.

Referees positioning and distance to the ball have consistently been considered decisive variables to support soccer referees’ accurate judgment and decision-making [2,8,11,14]. In fact, a wrong positioning from the referee may lead to incorrect decisions [1,8,14]. To decrease the rate of wrong decisions, FIFA advised referees to displace in the field by using preferentially the diagonal line that crosses the center of the field concerning the pitch corners, while also avoiding being ahead of the ball [30]. The present case study explored a new metric that supports the understanding of the referee’s diagonal displacements by considering the heat map and ellipsis area of displacements. This new metric enables measuring and understanding the predominance of the referee’s diagonal displacements according to, for example, the match’s difficulty or the teams’ style of play. Such analysis could help to analyze referees’ performance to ensure the appropriate positioning and game management (game reading, knowing where to stand, and adaptability) [31].

These diagonal displacements are relevant since the distance to the ball seems to play a critical role in the referee’s decision-making [1,8,12]. In the present case study, the average values of distance to the ball were ~18 m. The literature has provided inconclusive results regarding the relevant distances that would enhance the referees’ likelihood of making the right decisions. Some authors reported that more accurate decisions are made between 11 and 25 m [1,8], whereas others argued that referees are more capable of making the right decisions when showing a lower distance (i.e., 10 m) [12]. Despite that, the English Football Association proposed that the referees should not be more than 20 m further from the ball location [32], as greater distances may not allow the referees to perceive the playing situations properly, and consequently increasing the error rate [12,33]. In this study, the values were within this limit, suggesting that the ball location has a key role in guiding the referee’s positioning to ensure that he can identify the relevant information that might lead him to whistle or not [34].

We found that ~50% of the referee distances vary according to the ball location, reflecting the dynamic and unpredictable nature of the game. While the referee may be in the right place to judge a playing situation, it may be possible that a sudden change in ball possession may afford the team that gets ball possession to play a long ball for a counter-attack situation. Though, it may also reflect the areas that the referees usually cover during the game. While in the central zones of the pitch, the referees are more likely to present closer distances to the playing situation, but when the ball is close to the lateral sides, the referees may adjust their positioning according to the assistant referees, and consequently, present higher distances (~22 m) [8]. Further research should be developed to understand how such distances vary according to ball position on the field and, for example, concerning the center of the displacements’ ellipsis area.

Overall, the assistant referees’ role is to support the main referee decisions, mainly to the offside rule. Similarly, the assistant referees’ positioning is likely to affect their decisions. In fact, the key factors related to correct decisions of assistant referees are the angle of view and the distance to the offside line [13], as they need to perceive both the player in ball possession, the potential receiver, and the 2nd last defending player position while also being able to be aligned with the 2nd last defender. Therefore, it is clear that the assistant referees should adequately follow the offside line. In this respect, the assistant referees were found to be in line with the offside line when showing a position of less than 0.20 m from the offside line [13]. In the current study, the difference between the assistant referee and the 2nd last defender was ~1 m, which may support the assumption that the assistant referees trail (~53%) or lead (~33%) offside line [8]. Further research should be developed to understand if using the current method would distinguish differences in the distance between the assistant referee and the 2nd last defender for correct and wrong decisions of offside, or even according to a different type of passes (short or long passes). Such analysis could allow developing more appropriate training tasks for assistant referees and identify, over the training process, the variations on each referee’s distances to the offside line.

Regarding the analysis between halves, differences were observed in the assistant referees’ distance to the offside line. While the second assistant referee presented lower values of alignment with the offside line during the 1st half, during the 2nd half, the opposite has been detected (i.e., the first assistant referee showed the lower distance to the offside line). Such results may be possibly related to differences in the teams playing style and not only changes in assistant referees performance to maintain the positioning according to the playing time. That is, during the 1st half, the second assistant referee presented higher distances to the offside line, which may be linked with controlling offsides from a team adopting a counter-attack playing style that may promote more misalignments between the attackers, the defenders, and the assistant referee. In contrast, during the 2nd half, one assistant referee increased the distance to the offside line, while the other assistant referee decreased it. Accordingly, it may be possible that during the 2nd half, the second assistant referee had to control the offside from a team adopting a more positional play that might allow him to maintain closer distances to the offside line. This variable seems to be sensitive to capture the assistant referees positioning variability due to the teams playing style. These assumptions require clarification, so further studies may explore how different playing styles affect assistant referees’ distance to the offside line. Moreover, future studies should also be developed to design and test new metrics that explore the coordination tendencies between referees and assistant referees over the game and according to the ball’s positioning. This information may provide valuable knowledge about the accuracy of soccer referees judgment and decision-making process.

## 5. Conclusions

The results from this case study suggested that the proposed measures may be used to understand the positioning of soccer elite referees during match-play. The elite referee maintained a distance of ~18 m to the ball location in the observed match, mainly in a diagonal perspective, as revealed by his heatmap and ellipsis area. Regarding the assistant referees, average values of 1 m distance to the offside line were detected, suggesting that assistant referees attempt to maintain short distances to the offside line. The difference between the assistant referees from the 1st to the 2nd half may show sensitivity to the teams playing style and the game’s dynamic and unpredictable nature. Considering this, it may be possible that the present variables are sensitive to capture the adaptations in the referees positioning as the match progresses. Nevertheless, it is important to note that the results were sustained on a case study, and thus, it may refrain from achieving more robust inferences. Despite that, relevant and practical information can be depicted, which might help induce variability and adaptation in specific practice planning of soccer referees.

## Figures and Tables

**Table 1:** Characterization of the distance from the referee to the ball.

Descriptive Statistics	1st Half	2nd Half
Mean (m)	18.67	19.16
Median (m)	17.66	18.25
Coefficient of variation (%)	50.34	50.79
Skewness (a.u.)	0.48	0.59
Maximum (m)	56.30	55.79

## Data Availability

Not applicable.
